# Do we want more cancer patients on clinical trials If so, what are the barriers to greater accrual

**DOI:** 10.1186/1745-6215-9-31

**Published:** 2008-06-03

**Authors:** Andrew J Vickers

**Affiliations:** 1 Memorial Sloan-Kettering Cancer Center, 1275 York Avenue, NY, NY 10021, USA

## Abstract

It is often stated that only a small proportion of adult cancer patients participate in clinical trials. This is said to be a bad thing, with calls for more trials to include more patients. Here I argue that whether or not greater accrual to clinical trials would be a good thing depends on the trials we conduct. The vast majority of clinical trials in cancer are currently early phase trials, and most do not lead to further studies even if they have encouraging results. The key metric is thus not the number of patients on clinical trials, but the number on the sort of large, randomized, Phase III trials that can be used as a basis for clinical decisions. I also address two important barriers to greater clinical trial participation. The first barrier is financial: clinical research has long been the poor cousin of basic research, with perhaps no more than a nickel in the cancer research dollar going to clinical research. The second barrier is regulatory: clinical research has become so overburdened by regulation that it takes years to initiate a trial, and dedicated staff just to deal with the paperwork once the trial starts. This not only adds significantly to the costs of clinical research, but scares many young investigators away. It has been estimated that nearly half of all US-sponsored trials are being conducted abroad, and it is plausible that excessive regulation is at least partly responsible. That statistic should serve as a wake-up call to the US clinical research community to implement the recommendations of the now decade-old report of National Cancer Institute Clinical Trials Program Review Group, which largely center around simplifying trials and streamlining trial procedures.

## Background

In a recent paper in *Trials*, Wright et al examined divergent understandings of what constitutes a clinical trial in oncology[1]. The motivation for their study was to inform statistics and debates about clinical trial participation. It is often stated that only a small proportion of adult cancer patients participate in clinical trials – less than 5% being a typical estimate[2] – and that this needs to increase. Wright et al argue that it might be difficult to define exactly how many patients are or should be on clinical trials if there is no clear agreement on what qualifies as a trial.

In this editorial, I will discuss two related questions. First, should we want more cancer patients on clinical trials? Second, what are the practical steps we should take to ensure better clinical trial accrual? My thoughts on these questions are very much a personal perspective from my own experience of working in a major cancer center in the USA. Although other authors have drawn similar conclusions[3,4], it is for the reader to judge the degree to which they are applicable to other countries, or to non-academic settings.

## Discussion

### Do we want more cancer patients on clinical trials?

It is often assumed that higher clinical trial accrual would automatically be a good thing; certainly for medical research and future patients, but likely also for those currently being treated for cancer. But whether or not greater accrual to clinical trials would be of benefit depends on the trials we conduct. Putting more cancer patients on trials is just a means to an end – better data to inform clinical care – and we have to keep the end primary. As Doug Altman remarked, we do not need more research, "we need less research, better research, research done for the right reasons"[5].

Unfortunately, all too many cancer clinical trials are done for the wrong reasons. Ian Tannock and colleagues at the Princess Margaret Hospital in Toronto recently surveyed investigators of Phase II trials. The purpose of Phase II trials is to determine whether there is sufficient preliminary evidence of efficacy to warrant a large, randomized Phase III trial as a definitive test of whether a cancer agent is of value. Tannock contacted investigators of 36 Phase II trials with promising results and found that only 10 intended to conduct a subsequent Phase III. He concluded that "the real purpose of many phase II trials may be to legitimize the use of nonapproved compounds, or to enhance the investigators' CVs"[6].

I recently estimated that there are at least 12 times as many early phase trials in cancer as randomized Phase III trials[7]. Such trials have their place – indeed, they are essential to drug development – but they cannot usually help an oncologist make a decision about clinical care. "Getting more patients on trials" will therefore not improve cancer outcomes if the trials are all early phase, and especially if the trials are not intended to prompt further research. The key metric, I suggest, is not the number of patients on clinical trials, but the number on the sort of large, randomized, Phase III trials that can be used as a basis for clinical decisions[8]. Not all Phase III trials will be good ones, and not all will ask important questions, but if we had to choose one number to reflect our progress, the number of patients on Phase III trials would be it.

### How do we get more patients on clinical trials?

There are many barriers to increasing clinical trial enrollment; I will focus on just two, financing and regulation. With respect to funding, I have little doubt that clinical research is the poor relation in cancer research. For example, the funding of co-operative groups, which organize most of the large randomized trials in cancer, has been flat for years and constitutes only $150 m of the National Cancer Institute's $5 bn budget[9]

Any attempts to expand clinical trials in cancer will also need to address the issue of regulatory burden. Dilts et al have estimated that initiating a clinical trial through CALGB, one of the major US groups organizing large cancer trials, typically requires more than two years and over 370 separate steps[10]. Even a small, single-center trial can be a huge burden to initiate, requiring review by multiple clinical, scientific and ethical review committees. Many investigators describe the approval process for clinical trials as "traumatic" or "nightmarish" and I have met several young, smart, savvy investigators who have told me that they would love to do clinical trials, but "can't face" going through the approval process and, as a result, are going to stick to laboratory work and retrospective studies. Of course, it is only once a trial gets going that the regulatory burden *really *kicks in. Paperwork and documentation requirements for a typical cancer trial are so intense that, at our hospital, we typically allocate one full-time research assistant for 50 trial patients per year. In other words, it takes about an entire work week to manage the paperwork of a single clinical trial patient. It is true that, in some cases, one might lay at least some of the blame for excessive paperwork on poor design and unnecessary data collection; nonetheless, one cannot help but speculate as to how much researcher time might be freed up were regulatory requirements to be liberalized.

The National Cancer Institute is not unaware of these problems. A "Clinical Trials Program Review Group" has put together an excellent report[11], some of my favorite quotes from which are given in table 1. But note one thing: the report is over 10 years old. It is all very well for leading experts to call for simplifying the informed consent process, reducing eligibility criteria and limiting the number of endpoints, but it is as good as useless if these recommendations are not taken up by those who write or review protocols.

**Table 1:** 

Excerpts from the Report of the *National Cancer Institute Clinical Trials Program Review Group August 26, 1997*

*Informed Consent:*
It is the opinion of the Review Group that the informed consent process is onerous and overly cautious. In many cases it has become a disclaimer for institutions rather than information for the participant. As a result, true informed consent is not being obtained and the informed process itself may be inappropriately deterring individuals from participating in clinical trials ... Simplified informed consent documents will assist both trial participants and physicians ... and are essential.

*Trial eligibility criteria*
[There are] far too many exclusion criteria in the current clinical trials system. Potential enrollees are disqualified for seemingly arbitrary reasons from trials for which they would otherwise qualify ... The eligibility criteria for all cancer clinical trials should be simplified in order to require minimal input at the time of registration of individuals, and to substantially reduce the workload for the individual conducting the registration.

*Protocol development*
Rapid protocol development is critical to the ability to implement new ideas and concepts in an expeditious fashion. Groups should develop a common algorithm for protocol development in order to minimize the time necessary to develop and obtain a letter of intent or concept to NCI for consideration and review.

*Data collection*
Data collection should be reduced so that only data pertinent to the study endpoints and patient safety are accrued. In addition, NCI-funded efforts should include some large, uncomplicated trials in common cancers with minimal data requirements and accrual goals large enough to establish treatment differences definitively.

*Use of information technology to facilitate clinical trials management*
Forms required for trial randomization do not take advantage of computer systems creating even more work for the investigator. The resulting enrollment system is slow, inefficient and costly. The money that could be saved through a more uniform and streamlined process could be used to enroll more patients in trials. A single informatics system for the NCI, all cancer centers, and all cooperative groups is important to the success of the clinical trials program.

## Conclusion

The most commonly diagnosed cancer in men is prostate cancer. Yet at the time of writing there have been no adequate trials comparing the two major treatment approaches, radiotherapy and surgery[12]. It is obvious to me that we should prefer a single 10,000 patient randomized trial comparing primary treatment for prostate cancer to having 20,000 patients join the sort of early phase trials that typically go nowhere. What we need is not more patients on clinical trials, but more patients on the right sorts of trials.

Giving the barriers I have discussed above, getting these trials done will not be straightforward. Indeed, many US-based clinical researchers have simply given up and are conducting research abroad: one estimate is that about 45% of US-sponsored trials are currently being conducted overseas[13]. This statistic should serve as a wake-up call to the cancer research community: those billions of dollars we spend on laboratory research will only have been a good investment if new therapies or tests discovered in the laboratory turn out to improve patient care, and that will only happen if we do the right clinical trials.

## Competing interests

The author declares that he has no competing interests.

## Authors' contributions

AV conceived the article based on an idea by AV, AV wrote the article and reviewed it for important intellectual content. Financial and administrative support was provided by AV, AV read and approved the final manuscript.
